# Isolation and genomic characterization of SfI, a serotype-converting bacteriophage of *Shigella flexneri*

**DOI:** 10.1186/1471-2180-13-39

**Published:** 2013-02-17

**Authors:** Qiangzheng Sun, Ruiting Lan, Yiting Wang, Jianping Wang, Yan Wang, Peijing Li, Pengcheng Du, Jianguo Xu

**Affiliations:** 1 State Key Laboratory for Infectious Disease Prevention and Control, National Institute for Communicable Disease Control and Prevention, China CDC, P.O. Box 5, Beijing, Changping, China; 2 School of Biotechnology and Biomolecular Sciences, University of New South Wales, 2052, Sydney, NSW, Australia

## Abstract

**Background:**

All *Shigella flexneri* serotypes except serotype 6 share a common O-antigen tetrasaccharide backbone and nearly all variations between serotypes are due to glucosyl and/or O-acetyl modifications of the common O unit mediated by glycosyltransferases encoded by serotype-converting bacteriophages. Several *S. flexneri* serotype-converting phages including SfV, SfX, Sf6 and SfII have been isolated and characterized. However, *S. flexneri* serotype-converting phage SfI which encodes a type I modification of serotype 1 (1a, 1b, 1c and 1d) had not yet been characterized.

**Results:**

The SfI phage was induced and purified from a *S. flexneri* serotype 1a clinical strain 019. Electron microscopy showed that the SfI phage has a hexagonal head and a long contractile tail, characteristic of the members of *Myoviridae* family. SfI can convert serotype Y to serotype 1a and serotype X to serotype 1d, but cannot convert 10 other *S. flexneri* serotypes (1a, 1b, 2a, 2b, 3a, 3b, 4a, 4b, 5a, Xv) tested, suggesting that SfI has a narrow host range. Similar to other *S. flexneri* serotype-converting phages, SfI integrates into the *tRNA-thrW* gene adjacent to *proA* of the host chromosome when lysogenized. The complete sequence of the SfI genome was 38,389 bp, encoding 66 open reading frames and two tRNA genes. Phage SfI shares significant homology with *S. flexneri* phage SfV, *Escherichia coli* prophage e14 and lambda, and is classified into the lambdoid phage family. SfI was found to use a *cos* mechanism for DNA packaging similar to that of phage SfV.

**Conclusions:**

SfI contains features of lambdoid phages and is closely related to *S. flexneri* phage SfV, *E. coli* prophage e14 and lambda. The characterization of SfI enhances our understanding of serotype conversion of *S. flexneri*.

## Background

*Shigella* is the major cause of endemic bacillary dysentery (shigellosis) in developing countries. It is estimated that there are about 164.7 million cases of shigellosis annually worldwide, of which 163.2 million were in developing countries, resulting in 1,1 million deaths, most of which were children under 5 years of age [1]. Among the four *Shigella* species, *S. dysenteriae*, *S. flexneri*, *S. boydii*, and *S. sonnei*, *S. flexneri* is the predominant species.

Based on the combination of antigenic determinants present in the O-antigen of the cell envelope lipopolysaccharide (LPS), *S. flexneri* is further divided into various serotypes. To date, at least 16 serotypes have been recognized [2-4]. Except for serotype 6, all share a basic repeating tetrasaccharide unit, comprised of one GlcNAc and three rhamnoses [4]. Modifications to the side chain of the tetrasaccharide by the addition of glucosyl and/or *O*-acetyl groups give rise to various antigenic determinants [3]. The genes responsible for the O-antigen modification are always either the gene cluster *gtrABC* for glucosyl groups or the single *oac* gene for the *O*-acetyl group; all encoded by serotype-converting bacteriophages [3,5-10]. In all glucosylation modification phages, the *gtrABC* gene cluster is always located immediately upstream of the *attP* site, followed by the *int* and *xis* genes [6].

Up to now, four *S. flexneri* serotype-converting bacteriophages, SfV, SfX, Sf6 and SfII, have been induced and purified by different groups [8,11-13]. Morphologically, SfV and SfII, which have an isometric head and a long tail, belong to Group A in the family of *Myoviridae*[8,11]; while SfX and Sf6, which possess a short tail linked to an isometric head, belong to the family of *Podovirida*[12,13]. The complete genome sequences of phage SfV and Sf6 have been obtained by directly sequencing the phage DNA purified from phage particles, and their genetic features have been well characterized [9,10]. Recently, the prophage genome of SfX was determined from the sequenced *S. flexneri* serotype Xv strain 2002017; which is presumably the whole genome of phage SfX, because a SfX phage particle can be induced and isolated from 2002017 [2]. The SfX genome is 37,355 bp length, encoding 59 ORFs (unpublished data). The genome of SfII has not yet been sequenced from free phage particles, but prophage genomes can be derived from sequenced *S. flexneri* serotype 2a strains Sf301 and 2457T [14,15], which show considerable variation with one or both being prophage remnants.

*S. flexneri* serotype 1 is defined by reaction with type I antisera. A total of 4 subtypes, 1a, 1b, 1c and 1d have been recognized [16-18]. In serotype 1, a glucosyl group is attached to the GlcNac residue of the repeating unit by an alpha-1, 4 linkage, which results in the presence of serotype 1-specific I antigen. The type I modification is mediated by an O-antigen glucosylation locus (*gtrI*, *gtrA*, *gtrB*) encoded on the SfI prophage genome [5]. The glucosylation genes and flanking partial SfI sequences were previously obtained from a serotype 1a strain Y53 [17]. However, the free phage particle of SfI had not been isolated, and its full genomic characteristics have not yet been elucidated [5].

In this study, we induced and purified the free SfI phage particles from *S. flexneri* serotype 1a clinical strain 019 and characterized its morphology, host range and genomic features.

## Results and discussion

### Isolation of phage SfI from *S. flexneri* serotype 1a strain 019

Using the conditions described in Methods, we induced the SfI phage from serotype 1a strain 019. Plaques were observed on the semi-solid LB agar when the host strain 036 was infected with induced products from strain 019. Lysogens isolated from plaques were serologically identified as serotype 1a, characterized by agglutination with both typing sera I and grouping sera 3;4. PCR amplification indicated that the SfI specific gene *gtrI* is present on both phage particles and the lysogens. These results suggest that phage SfI has been successfully induced and isolated from strain 019. This is the first report of isolation of free SfI particles from *S. flexneri*.

### The morphology of SfI is characteristic of the *Myoviridae* family

**Figure 1:**
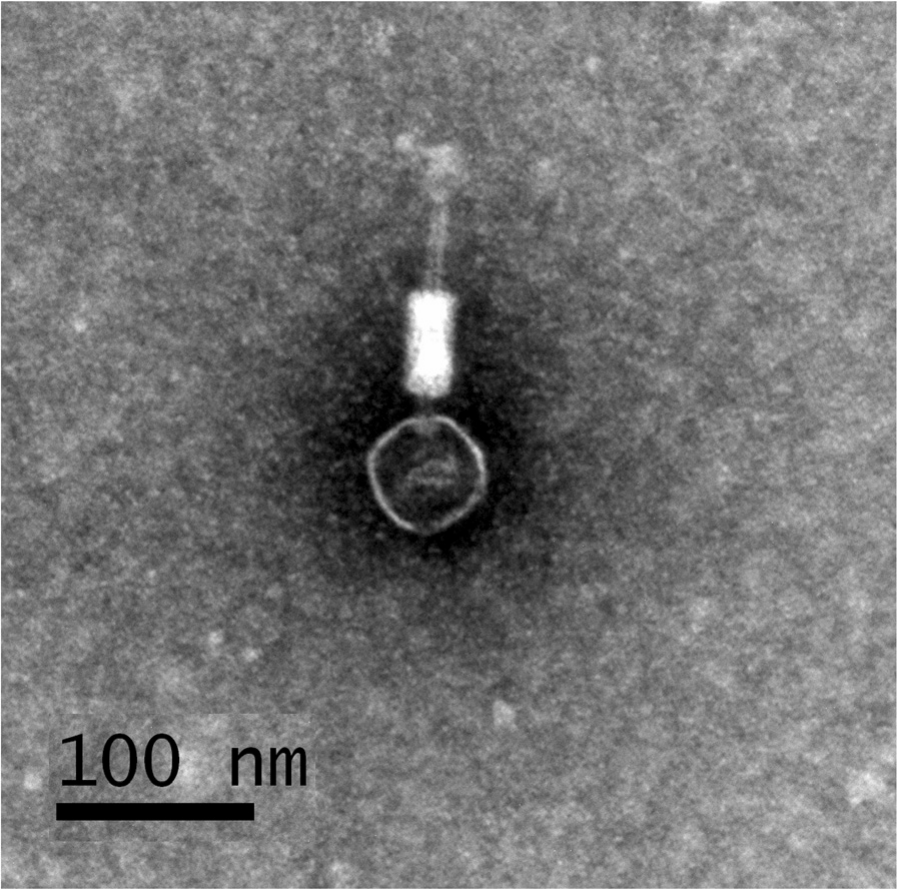
**Electron micrograph of *S. flexneri *bacteriophage SfI stained with phosphotungstic acid.**

The purified SfI phage particles were morphologically analyzed using electron microscopy. The phage has a hexagonal head of ca. 55 nm in diameter, a knob-like neck, a contractile tail of ca. 110 nm, and a tail sheath of ca. 55 nm (Figure 1). There are indications of a baseplate-like structure and long tail fibers, but no other distinctive features could be seen (Figure 1). These characteristics suggest that phage SfI is a member of the *Myoviridae* family in the order *Caudovirale*[19].

**Figure 2:**
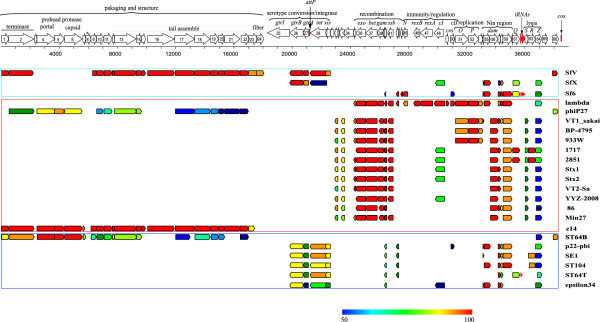
**Genetic map of *S. flexneri *bacteriophage SfI and comparison of SfI with related phages and prophages.** The SfI genome is shown to scale. Numbers below the scale bar are the number of base pairs. Arrows above the scale represent the predicted genes and orientation. ORFs were shown within the boxes, and putative function modules and names of genes are given above. Vertical black lines indicate *cos* and *attP* sites respectively. Red arrows indicate tRNA genes. Pseudogenes are marked with a black asterisk. Below the scale, arrows represent homologous proteins of bacteriophages and prophages from different hosts with *S. flexneri*, *E. coli* and *Salmonella* framed within a green, red or blue box, respectively. Homologs between SfI and other phages/prophages are shown in different colors with color coding corresponding to level of homology at amino acid level, with red of 100% identity and blue of > =50% identity.

In comparison to other morphologically characterized serotype-converting phages Sf6, SfV, SfII and SfX, SfI has a very similar appearance to SfII and SfV [8,11], but distinctive from SfX and Sf6 [12,20]. The microscopic difference reflected the genetic divergence among them in that the SfI packaging and structure genes were identical to those of phage SfV, but divergent from those of SfX and Sf6 (see below, Figure 2).

### Phage SfI has a very narrow host range

Host specificity of serotype-converting bacteriophages has long been recognized, which results in the specific lytic spectrum and serotype conversion of *S. flexneri* in nature [20]. The recognition between the O-antigen of host bacterium and the tail component of a phage is the key mechanism of host specificity [20]. To determine the host range of SfI, 132 *S. flexneri* strains of 12 serotypes (1a, 1b, 2a, 2b, 3a, 3b, 4a, 4b, 5a, Y, X and Xv) were tested following the methods described in the Methods. Apart from 10 serotype Y strains, which were all converted to serotype 1a as expected, the 24 serotype X strains tested were also lysogenized, and converted to a newly named serotype 1d [16]. The serotype 1d strains were serologically characterized as reacting with both serotype 1 specific I typing sera and serotype X specific 7;8 grouping sera [16]. Interestingly, such a serotype has already appeared in natural infections in Anhui and Henan provinces, China [21]. Except for serotypes Y and X, the other serotypes could not be lysogenized by phage SfI. A possible explanation for the host range restriction of phage SfI is phage immunity due to modification of the O-antigen as phage receptors [22].

### SfI uses a site-specific mechanism for DNA packaging and has the same *attP* core sequence as SfII, SfIV, SfV and SfX

Restriction enzyme analysis revealed that phage SfI has a linear but not circular genome (data not shown). Genomic comparison found that the SfI prophage genome has similar packaging genes to that of phage SfV; and the fragments adjacent to them were also highly similar to the cohesive end site (*cos*) of phage SfV [9], with only one base difference at the 5^′^ end (T versus A). These data suggest that SfI may use the same site-specific mechanism as SfV for packaging. Direct sequencing of the putative termini of the SfI genome extracted from free phage particles and comparison of the corresponding regions with the SfI prophage genome in strain 019 revealed a 10 nucleotide (5^′^- TGCCCGCCCC -3^′^) gap in the SfI phage genome. Therefore, we conclude that SfI uses a *cos* mechanism for DNA packaging as postulated for phage SfV [9], and does not use a head full mechanism (*pac*) as for phage Sf6 and SfX [10,12].

**Figure 3:**
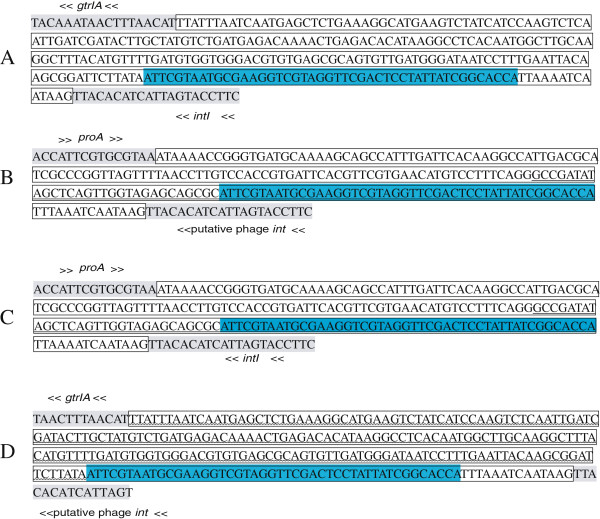
**DNA sequences of chromosomal integration site of *S. flexneri *phage SfI.** Sequences obtained by PCR and sequencing of junction regions using a series of primers across the integration site. (**A**) *attP* in phage SfI. (**B**) *attB* in strain 036. (**C**) *attL* in strain 036_1a. (**D**) *attR* in 036_1a. Sequences in box are DNA regions between conserved genes; Underlined sequences are *tRNA-thrW*; Sequences in blue are *att* core sequence; Conserved genes are shaded and their transcription orientation is marked by an arrow.

Integration of lambdoid phages into the bacterial chromosome generally occurs by site-specific recombination between the phage *attP* and the bacterial *attB* sites [23]. In all serotype-converting phages except for Sf6, the *attP* site is always found located immediately downstream of the O-antigen modification genes, and preceded by the *int* and *xis* genes [6]. To determine the *attP* site of phage SfI, the region between genes *gtrA* and *intI* of SfI was PCR amplified and sequenced and a 261 bp sequence was obtained, in which, 46 bases, ATTCGTAATGCGAAGGTCGTAGGTTCGACTCCTATTATCGGCACCA, were found to be identical to the *attR/attL* core sequence of prophage SfI in strain Y53 [5] (Figure 3). In the lysogen of 036_1a, the 261 nucleotide sequence was divided into two parts, located at opposite ends of the SfI prophage genome (Figure 3). Evidently, site-specific recombination occurred at this *attP* site. The *attP* core sequence of SfI is identical to that of *S. flexneri* serotype-converting phage SfII, SfV and SfX, as well as that of serotype-converting phages p22 of *Salmonella typhimurium* and DLP12 of *E. coli*[5,8,24].

### Characterization of SfI genome sequence

The complete genome sequence of SfI was obtained by combining the SfI prophage genome of host strain 019 with the *attP* site obtained by PCR sequencing as above. Firstly, the whole genome sequence of host strain 019 was sequenced using Illumina Solexa sequencing. A total of 4,382,674 reads were generated to reach about 110-fold coverage and assembled *de novo* into 376 contigs and scaffolds. The SfI prophage genome located between genes *int* and *gtrIA* was extracted from one of the contigs which was further assembled with the *attP* site sequence obtained above to construct a circular phage SfI genome. To revert to the linear organization as usual practice, we artificially linearised the sequence starting from the terminase small subunit gene and ending with the *cos* site (Figure 2).

The genome size of SfI is 38,389 bp similar to that of sequenced *S. flexneri* serotype-converting phages Sf6 (39,043 bp) [9], SfV (37,074 bp) [10] and SfX (37,355) (unpublished data). The overall G + C content is 50.12%, which is very similar to that of its host (50.9%) [25]. Sixty-six putative ORFs (including one pseudogene) were predicted and their functions are listed in the Additional file 1: Table S1.

The genetic architecture of the SfI genome is similar to that of sequenced *S. flexneri* serotype-converting phages SfV, Sf6 and SfX: the left-most region encodes genes for phage packaging and structure, followed by the middle region with genes involved in serotype conversion, integration/excision, recombination, immunity and regulation, replication and the Nin region, and then the putative lysis cassette at the right-most region ending with the *cos* site of the phage genome (Figure 2). The genomic structure of SfI is also similar to that of phage SfV and lambda. Thus it belongs to the family of lambdoid phages.

tRNAscan was used to find tRNA genes. Two tRNA genes in tandem, with anticodons GUU for asparagine (Asn) and UGU for threonine (Thr), were found to be located downstream of gene Q (35,738 – 35,809 for Asn, and 35,818 – 35,890 for Thr). One or both of these tRNA genes were also to be found located at this position in phage Sf6, ST64T, PS3 and p21 [10,26,27]. A recent study suggested that phage-encoded tRNA could serve to supplement the host tRNA reservoir, allowing the rare codons in the phage to be more efficiently decoded [28]. Codon analysis indeed found a convincing bias of ACA (anticodon UGU) in the SfI genome when compared to its *S. flexneri* host (with 17.3% in phage SfI, and 7.1% in strain Sf301), but no obvious bias was observed on CAA (anticodon GUU), and the significance of the *tRNA-Asn* in SfI is not clear.

### Genomic comparison reveals that SfI is genetically related to *Shigella* phage SfV, *E. coli* prophage e14 and lambda

The ORFs encoded in the SfI genome were searched against the GenBank database at both DNA and amino acid levels. SfI encoded proteins exhibited homology to various phages and prophages originating from various hosts, including *Shigella* (SfV, Sf6 and SfX), *E. coli* (lambda, phip27, VT1-sakai, BP-4795, 933 W, 1717, 2851, Stx1, Stx2, VT2-Sa, YYZ-2008, 86, M27 and e14) and *Salmonella* (ST64B, p22-pbi, SE1, ST104, ST64T and epsilon34). Figure 2 displays the homologies of phage SfI to other phages. The SfI genes involved in phage packaging and morphogenesis are homologous and organized in a similar manner to those of phage SfV, phi-p27, ST64B and prophage e14. As reported earlier [6], the O- antigen modification and integration and excision modules (*gtrA, gtrB, int* and *xis*) are homologous to that of serotype-converting bacteriophages from *S. flexneri* (SfV and SfX) and *Salmonella* (p22-pbi, SE1, ST104, ST64T and epsilon34). However, the early and regulatory regions located in the right half of the genome were homologous to that of lambda and Shiga toxin-1 and Shiga toxin−2 phages (phip27, VT1-sakai, BP-4795, 933 W, 1717, 2851, Stx1, Stx2, VT2-Sa, YYZ-2008, 86 and M27).

Therefore SfI is a mosaic phage with its left half most homologous to phage SfV (91.6% - 100% identity at protein level, and 89-98% at DNA level [ORF by ORF comparison]) and *E. coli* prophage e14 (94.0% - 100% identity at protein level, and 97% at DNA level) and right half most homologous to Lambda (67% - 100% identity at protein level, and 80 - 98% at DNA level). Homology to SfV encompasses at least 23 ORFs encoding functions for morphology (*orf1*, *orf2* and *orf9* - *orf24*), O-antigen modification (*orf26*, *orf27*), integration/excision (*orf28* to *orf29*) (Figure 2, Table 1). The homologous ORFs are located in four contiguous regions, amounting to 17,487 bp nucleotides and accounting for 45.6% of the entire phage genome (Table 1). SfI also shared genetic relatedness with the *E. coli* prophage e14. The homologous regions mainly encode proteins responsible for phage assembly and morphogenesis and are located in the left half of the SfI genome (Figure 2 and Table 1). The homologous regions account for 46% of the SfI genome. Based on the homology of the first 22 ORFs (Additional file 2: Figure S1), it seems that SfI is closer to e14 than to SfV since 5 ORFs (SfI *orf3* to *orf7*) are highly homologous between SfI and e14, but share little homology between SfI and SfV. For the remaining 17 ORFs except *orf8*, the pairwise percentage identities are very similar between SfI, SfV and e14. On the other hand, the homology between SfI and SfV extends further to *orf28* with high homology of *orf23*, *orf24* and *orf26* to *orf28*. Similarly, six contiguous DNA segments, which account for 28.4% of the SfI genome, were found to be homologous to the corresponding regions of lambda. These homologous regions are mainly located in the early and regulatory regions, and encode functional modules for phage recombination (*orf35* to *orf4*3), immunity and regulation (*orf45* to *orf50*), replication (*orf51*, *orf52*), Nin region (*orf53* to *orf55*, *orf57* to *orf60*), and part of the lysis module (*orf64*) (Figure 2 and Table 1). Thus a total of 72.9% of the SfI genome is homologous to either SfV, e14 or lambda.

**Table 1:** **Homology of SfI to *S. flexneri *phage SfV and *E. coli *prophage e14 and lambda**

**Phage or prophage**	**Nucleotide position**	**Homologous nucleotide position in SfI (total length [bp])**	**% identity at nucleotide level**	**SfI ORFs**^***a***^	**% of SfI genome**
SfV	9 – 2,211	2 – 2,194 (2,193)	98	*orf1*, (*orf2*)	45.6
	5,793 – 17,782	6,053 – 18,042 (11,990)	97	*orf9 - orf24*	
	19,146 – 22,042	19,787 – 22,681 (2,895)	98	(*orf26*), *orf2 - orf29*, *attP*	
	36,666 – 37,074	37,964 – 38,372 (409)	89	(*orf66*)	
Lambda	30,418 – 30,910	23,002 – 23,493 (491)	95	(*orf31*), *orf32*, (*orf33*)	28.4
	31,206 – 34,381	24,281 – 27,456 (3,176)	98	(*orf35*), *orf36 - orf43*	
	35,104 – 35,386	27,708 – 27,990 (283)	98	(*orf45*)	
	35,496 – 41,084	28,052 – 33,640 (5,590)	98	*orf46 - orf55*	
	42,097 – 43,068	2 - 2,194 (2,193)	97	*orf57 - orf59*, (*orf60*)	
	45,966 – 46,361	6,053 -18,042 (11,990)	80	(*orf64*)	
e14	2,840,259 - 2,859,298^*b*^	1 - 17,234, 36,721 - 38,389 (17,660)	97	*orf1 - orf22*, (*orf66*)	46%

^*a*^ Parentheses indicate that the region of homology starts or ends within an ORF.

^*b*^*E. coli* S88 strain genome (accession no. CU928161).

## Conclusions

The serotype-converting bacteriophage SfI was isolated from a *S. flexneri* serotype 1a strain. It had a narrow lytic pattern and converted only serotype Y to serotype 1a and serotype X to serotype 1d. Morphologically SfI is a member of the *Myoviridae* family in the order of *Caudovirale*. Genomic analysis revealed that SfI contains features of lambdoid phages and is closely related to *S. flexneri* phage SfV, *E. coli* prophage e14 and lambda. The characterization of serotype-converting phage SfI enhances our understanding of serotype conversion of *S. flexneri*.

## Methods

### Bacterial strains, media and culture

*S. flexneri* serotype 1a strain 019 [16] was used as the source for induction of phage SfI. *S. flexneri* strain 036 (serotype Y) was used as the host for phage infection and large volume propagation of SfI [16]. One hundred and thirty two *S. flexneri* strains of 12 serotypes (17 serotype 1a, 5 serotype 1b, 10 serotype 2a, 10 serotype 2b, 10 serotype 3a, 2 serotype 3b, 5 serotype 4a, 5 serotype 4b, 4 serotype 5a, 10 serotype Y, 24 serotype X and 30 serotype Xv) were used for phage host range detection. All *S. flexneri* strains used in this study were isolated from diarrheal patients in China, or purchased from National Collection of Type Cultures (NCTC), UK. *S. flexneri* strains were serologically identified using *Shigella* antisera Kits (Denka Seiken, Japan) and monoclonal antibody reagents (Reagensia AB, Sweden). *S. flexneri* strains were routinely cultured on LB agar or in LB broth with shaking at 37°C.

### Induction of phage SfI

Induction of phage SfI was performed as methods described by Mavris *et al.*[8]. Briefly, a freshly grown colony of strain 019 was incubated in 10 ml LB broth overnight with vigorous shaking. After being induced for 30 min at 56°C with aeration, the cultures were centrifuged, and the supernatants were filtered through a 0.22 mm membrane filter (Promega) to remove bacterial cells. The filtrates were either used directly for phage infection assay or stored at 4°C with addition of 10% (v/v) chloroform.

### Phage infection and lysogenization

*S. flexneri* strain 036 cells were prepared using the methods for phage lambda [29]. Phage infection and lysogenization were performed using the methods described previously [16]. The serotypes of isolated colonies were identified by slide agglutination assay. Large volume phage purification was performed on *S. flexneri* strain 036, according to the methods for phage SfII [8].

### Electron microscopy

The purified phages were absorbed on carbon-coated copper grids (300 mesh) and negatively stained with 2% (w/v) sodium phosphotungstate (pH 7.0). Samples were visualized with a Hitachi 600 electron microscope at 80 kV.

### Host range detection

To determine the host range of phage SfI, one hundred and thirty two *S. flexneri* strains of 12 serotypes were infected with SfI. The preparation of component cells, phage infection and lysogen isolation were performed as methods for strain 036 above. The SfI host range was determined by observing the presence of plaques and serologically identification of the lysogens.

### Identification of the chromosomal integration site and cohesive ends (*cos* sites) of phage SfI

Oligonucleotide primers *gtrI*-F (5^′^- ATTGAACGCCTCCTTGCTATGC -3^′^), *intI*-R (5^′^- AGTGTTACAGGAAATGGGAGGC -3^′^), *proA*-F (5^′^- ACAAAGCGAAATCATCCTCAA -3^′^), and *yaiC*-R (5^′^- GCAGGAAACCACCATCAACACC -3^′^), which are complementary to the genes *gtrI* and *intI* in phage SfI*,* and *proA* and *yaiC* in *S. flexneri* chromosome, respectively, were used to identify the *attP* and *attB* sites of phage SfI and strain 036, as well as the *attR* and *attL* regions of the SfI lysogen. PCR was conducted using the Sensoquest labcycler PCR System (SENSO, German) under standard protocol. The PCR products were either cloned into TA vector pMD20-T (TaKaRa) for sequencing or sequenced directly.

To determine the cohesive ends of the SfI phage, two primers, *cos*-F: 5^′^- ATGCCACCACGAACCCCAAAAG -3^′^ (nt 37,964 - 37,985, complementary to SfI genome sequence), *cos*-R: 5^′^- GGCTTGGGGCGACGCCCGGA -3^′^ (nt 72–91, complementary to SfI genome), were designed to sequence the putative termini of the SfI genome directly using phage DNA as the template. The phage genome ends obtained were further compared to the corresponding regions of the SfI prophage genome in strain 019. The missing region in the former sequence is the putative *cos* site of phage SfI.

### Genome sequencing and analysis

To obtain the entire phage genome sequence of SfI, the whole genome of source strain 019 was sequenced by Illumina Solexa sequencing. A paired-end (PE) library with an average insertion length of between 500 bp and 2,000 bp was constructed. Reads were generated with Illumina Solexa GA IIx (Illumina, San Diego, CA) and assembled into scaffolds using SOAP denovo (Release1.04). The sequence between genes *intI* and *gtrA* was extracted for further analysis. By assembling with the sequence amplified from SfI DNA using primer pair *gtrI*-F and *int*-R mentioned above, the entire sequence of SfI genome in its circular state was obtained. Open reading frames (ORFs) of SfI were determined using the ORF Finder program, which is accessible through the National Center for Biotechnology Information (NCBI). Searches for homologous DNA and protein sequences were conducted with the BLAST software against the non-redundant GenBank database (http://www.ncbi.nlm.nih.gov/blast/blast/). tRNA genes were determined with tRNAscan-SE Search server (http://lowelab.ucsc.edu/tRNAscan-SE).

### Nucleotide accession number

The genomic sequence of phage SfI has been deposited in GenBank as accession number JX509734.

## Competing interests

The authors declare that they have no competing interest.

## Authors' contributions

JX, QS and RL designed the study, and co-drafted the manuscript. YW participated in the induction of the phage. JW carried out the PCR amplification and DNA sequencing. PL participated in the phage induction and infection. YW and PD participated in the sequence alignment and genome annotation. All authors read and approved the final manuscript.

## Supplementary Material

Additional file 1: Table S1 Analysis of predicted ORFs and proteins of SfI. Click here for file

Additional file 2: Figure S1 Gene by gene comparison of homologous regions of SfI with *S. flexneri* phage SfV and *E. coli* prophage e14. The arrows indicate the predicted proteins and orientation of the ORFs. The regions marked with a lightly red rectangle represent >50% sequence identity at amino acid level. Click here for file
