# Supplementary material for: Isolation and genomic characterization of SfI, a serotype-converting bacteriophage of Shigella flexneri
Source: BMC Microbiol. 2013 Feb 17;13:39. doi: 10.1186/1471-2180-13-39 (PMC3636060; doi:10.1186/1471-2180-13-39)
Supplement: Additional file 1: Table S1 — Analysis of predicted ORFs and proteins of SfI. [file 1471-2180-13-39-S1.doc]

Additional file 1: Table S1 Analysis of predicted ORFs and proteins of SfI

| ORFs | Gene coordinate and orientation | Size of product | Putative function | Related phage and bacterial protein |  |  |
| --- | --- | --- | --- | --- | --- | --- |
|  |  |  |  | Proteins (size and origin) | Gene bank accession no | (%, Identities/AAs) |
| 1 | 51→545 | 164 | Small terminase  subunit | Terminase small subunit (164 aa, phage SfV) | NP_599033 | 6e-116, (99/164) |
| 2 | 542→2275 | 577 | Large terminase  subunit | Large terminase subunit (577 aa, phage SfV) | NP_599034 | 0.0, (98/563) |
| 3 | 2287→2469 | 60 | Unknown | Putative integral membrane protein (60 aa, phage ST64B) | NP_700376 | 5e-25, (97/60) |
| 4 | 2595→3710 | 371 | Portal protein | Portal Protein (414 aa, phage ST64B) | NP_700377 | 0.0, (99/371) |
| 5 | 3652→4338 | 228 | Pro-head protease | Pro-head protease (228aa, phage ST64B) | NP_700378 | 9e-164, (99/228) |
| 6 | 4353→5558 | 401 | Capsid protein | Major capsid protein precursor (401 aa, phage ST64B) | NP_700379 | 0.0, (97/40) |
| 7 | 5608→5808 | 66 | Unknown | Putative bacteriophage protein (66 aa, *E. coli* E22) | ZP_03045476 | 6e-39, (97/66) |
| 8 | 5811→6163 |  | Pseudo gene |  |  |  |
| 9 | 6160→6570 | 136 | Head-tail adaptor | Putative phage head-tail adaptor (136 aa, *E. coli* E22) | ZP_03045463 | 1e-98, (100/136) |
|  |  |  |  | Hypothetical protein SfVp07 (98 aa, phage SfV) | NP_599039 | 3e-66, (96/98) |
| 10 | 6545→7051 | 168 | Unknown | Hypothetical protein SfVp08 (168 aa, phage SfV) | NP_599040 | 4e-115, (98/168) |
| 11 | 7048→7608 | 186 | Unknown | Hypothetical protein SfVp09 (186 aa, phage SfV) | NP_599041 | 3e-134, (98/186) |
| 12 | 7617→7787 | 56 | Unknown | Hypothetical protein SfVp10 (56 aa, phage SfV) | NP_599042 | 6e-133, (100/56) |
| 13 | 7771→9267 | 498 | Tail sheath protein | Tail sheath protein (498 aa, phage SfV) | NP_599043 | 0.0, (99/498) |
| 14 | 9267→9623 | 118 | Tail tube protein | Hypothetical protein SfVp12 (118 aa, phage SfV)  Tail tube protein (118 aa, *E. coli* O157:H7) | NP_599044  ZP_02815453 | 2e-84, (100/118)  6e-84, (99/118) |
| 15 | 9623→9892 | 89 | Tail | Hypothetical protein SfVp13 (89 aa, phage SfV) | NP_599045 | 1e-59, (99/89) |
| 16 | 10034→11869 | 611 | Tail tape measure | Tail protein (611aa, phage SfV) | NP_599046 | 0.0, (99/611) |
| 17 | 11915→13258 | 447 | Tail/DNA  circulation protein | Tail/DNA circulation protein (447 aa, phage SfV) | NP_599047 | 0.0, (100/447) |
| 18 | 13255→14334 | 359 | Tail protein | Tail protein (359 aa, phage SfV) | NP_599048 | 0.0, (98/359) |
| 19 | 14334→14882 | 182 | Baseplate assembly | Tail protein (182 aa, phage SfV) | NP_599049 | 3e-132, (100/182) |
| 20 | 14882→15307 | 141 | Tail protein | Tail protein (141aa, phage SfV) | NP_599050 | 1e-98, (100/141) |
| 21 | 15294→16352 | 352 | Baseplate J-like  phage protein | Tail protein (352 aa, phage SfV) | NP_599051 | 0.0, (98/352) |
| 22 | 16343→16927 | 194 | Tail protein | Tail protein (194 aa, phage SfV) | NP_599052 | 1e-140, (98/194) |
| 23 | 16931→17581 | 216 | Tail fiber protein | Hypothetical protein SfVp21 (216 aa, phage SfV) | NP_599053 | 1e-141, (93/216) |
| 24 | 17490→17993 | 167 | Tail fibre assembly | Tail fibre assembly protein (167 aa, phage SfV) | NP_599054 | 2e-110, (92/166) |
| 25 | 18227←19747 | 506 | Glycosyltransferase | GtrI (506 aa, *S. flexneri*) | AAF09027 | 0.0, (100/506) |
| 26 | 19750←20670 | 306 | Bactoprenol glucosyl transferase | GtrBI (306 aa, *S. flexneri*) | AAF09026 | 0.0, (99/306) |
| 27 | 20667←21029 | 120 | Flippase | GtrAI (120 aa, *S. flexneri*) | AAF09025 | 3e-80, (99/120) |
| 28 | 21291←22454 | 387 | Integration | Integrase (387 aa, phage SfV) | NP_599058 | 0.0, (98/387) |
| 29 | 22331←22681 | 116 | Excisionase | Excisionase (147 aa, phage SfV) | NP_599059 | 4e-76, (94/116) |
| 30 | 22653←22931 | 92 | Unknown | Hypothetical protein ECBD_3120 (92 aa, *E.coli* BL21(DE3)) | YP_003037305 | 8e-63, (99/92) |
| 31 | 22979←23197 | 72 | Unknown | Conserved domain protein (72 aa, *E.coli* 101-1) | ZP_03070303 | 1e-46, (100/72) |
|  |  |  |  | Hypothetical protein PBV4795_ORF8 (73aa,Phage BP-4795) | YP_001449245 | 3e-42, (94/71) |
| 32 | 23204←23293 | 29 | Unknown | Hypothetical protein Stx2II_gp40 (29 aa,  Stx2 converting phage II) | YP_003828953 | 3e-12, (100/29) |
| 33 | 23296←23649 | 117 | Unknown | Hypothetical protein PBV4795_ORF9 (93 aa, Phage BP-4795) | YP_001449246 | 3e-41, (86/78) |
| 34 | 23519←24271 | 250 | Unknown | Hypothetical protein (250 aa, *E.coli* IAI39) | YP_002407962 | 0.0, (100/250) |
| 35 | 24268←24432 | 54 | Unknown | Hypothetical protein VT2-Sap12 (62 aa, phage VT2-Sakai) | NP_050511 | 8e-32, (96/54) |
|  |  |  |  | Hypothetical protein lambdap39 (60 aa, phage lambda) | NP_040615 | 1e-29, (94/60) |
| 36 | 24423←25103 | 226 | Recombination | Putative exonuclease (226 aa, Phage BP-4795) | YP_001449249 | 1e-170, (99/226) |
|  |  |  |  | Exonuclease (226 aa, phage lambda) | NP_040616 | 1e-168, (98/226) |
| 37 | 25100←26885 | 261 | Recombination | Bet protein (261 aa, phage 933W) | NP_049474 | 0.0, (100/261) |
|  |  |  |  | Recombination protein Bet (261 aa, Stx2-converting phage 86) | YP_794099 | 0.0, (99/261) |
|  |  |  |  | Bet (261 aa, phage lambda) | NP_040617 | 0.0, (99/261) |
| 38 | 25891←26307 | 138 | Host-nuclease inhibitor | Host-nuclease inhibitor protein Gam (138 aa, phage lambda) | NP_040618 | 5e-97, (97/138) |
| 39 | 26262←26531 | 89 | Host cell killing | Kil (89 aa, phage VT2-Sakai) | NP_050515 | 5e-60, (100/89) |
|  |  |  |  | Kil (89 aa, phage lambda) | CAA23979 | 1e-59, ( 99/89) |
| 40 | 26374←26538 | 54 | Regulatory | Antitermination protein (54 aa, phage lambda) | NP_040620 | 2e-33, (100/54) |
|  |  |  |  | cIII (54 aa, phage VT2-Sakai) | NP_050516. | 2e-33, (100/54) |
| 41 | 26611←26979 | 122 | Single-stranded  DNA binding | Putative single-stranded DNA binding protein (122 aa, phage lambda) | NP_040621 | 2e-85, (100/122) |
| 42 | 27182←27361 | 59 | Anti-restriction | Restriction alleviation protein (66 aa, phage lambda) | NP_040622 | 3e-38, (100/59) |
|  |  |  | Unknown | Ral (76 aa, phage phi21) | AAD15034 | 3e-38, (100/59) |
| 43 | 27345←27431 | 28 |  | Hypothetical protein lambdap47 (28 aa, phage lambda) | NP_040623 | 3e-10, (100/28) |
| 44 | 27486←27623 | 45 | Unknown | Gene 36 protein (45aa, phage Sf6) | NP_958212 | 8e-25, (98/45) |
| 45 | 27632←27964 | 110 | Antitermination | Gene 37 protein (110 aa, phage Sf6) | NP_958213 | 4e-74, (98/110) |
|  |  |  |  | N (133 aa, lambda) | AAA96578 | 8e-62, (92/106) |
| 46 | 28381←28815 | 144 | Exclusion | RexB,exclusion protein (144 aa, lambda) | NP_040626 | 3e-97, (100/144) |
| 47 | 28831←29670 | 279 | Exclusion | Exclusion protein (279 aa, phage lambda) | NP_040627 | 0.0, (100/279) |
| 48 | 29783←30496 | 237 | Repressor | *cI*, repressor (237 aa, phage lambda) | NP_040628 | 2e-176, (100/237) |
| 49 | 30597←30797 | 66 | Antirepressor | *Cro* (66 aa, phage lambda) | NP_040629 | 2e-42, (100/66) |
| 50 | 30916←31209 | 97 | Regulatary | *c*II protein (97 aa, phage lambda) | NP_040630 | 2e-64, (100/97) |
| 51 | 31242←32141 | 299 | Replication | DNA replication protein (299 aa, phage lambda) | NP_040631 | 0.0, (99/299) |
| 52 | 32138←32839 | 233 | Replication | DNA replication protein (233 aa, phage lambda) | NP_040632 | 5e-168, (97/233) |
|  |  |  |  | Putative replication protein P (233 aa, Phage BP-4795) | YP_001449264 | 6e-170, (98/233) |
| 53 | 32836←33126 | 96 | Exclusion | Putative Ren protein (96 aa, Phage BP-4795) | YP_001449265 | 4e-60, (93/96) |
|  |  |  |  | Ren exclusion protein (96 aa, phage lambda) | NP_040633 | 7e-45, (90/96） |
| 54 | 33123←33203 | 26 | Unknown | NinA protein (26 aa, Bacteriophage 21) | CAB39987 | 3e-09, (100/26) |
| 55 | 33200←33640 | 146 | Unknown | NinB (146 aa, phage lambda) | NP_040634 | 3e-103, (99/146) |
| 56 | 33607←34164 | 185 | DNA methylase | Similar to DNA methylase (175 aa, phage VT2-Sakai) | NP_050531 | 3e-127, (98/175) |
|  |  |  |  | Gp62 (175 aa, phage HK97) | NP_037744 | 6e-127, (98/175) |
| 57 | 34161←34343 | 60 | Unknown | Hypothetical protein HK620p28 (60 aa, phage HK620) | NP_112061 | 1e-36, (100/60) |
|  |  |  |  | Gene 52 protein (60 aa, phage Sf6) | NP_958226 | 1e-36, (100/60) |
| 58 | 34340←34510 | 56 | Unknown | NinF protein (56 aa, phage lambda) | NP_040638 | 2e-32, (96/56) |
|  | 34503←35114 |  |  | Gene 53 protein (56 aa, phage Sf6) | NP_958227 | 2e-32, (96/56) |
| 59 |  | 203 | Unknown | NinG protein (204 aa, bacteriophage 21) | CAB39991 | 4e-148, (100/203) |
|  |  |  |  | Gene 54 protein (203 aa, phage Sf6) | NP_958228 | 6e-148, (100/203) |
| 60 | 35111←35167 | 18 | Unknown | NinH protein (18 aa, bacteriophage 21) | CAB39992 | 2e-05, (100/18) |
| 61 | 35158←35646 | 162 | Antitermination | Late antiterminator (162 aa, bacteriophage Nil2) | CAC95104 | 8e-91, (98/162) |
|  |  |  |  | Antiterminator Q protein (162 aa，Stx2-converting phage 1717) | YP_002274250 | 3e-90,（98/162） |
| 62 | 36012←36227 | 71 | Lysis | Lysis protein S (71 aa, phage 21) | AAA32349 | 3e-45, (100/71) |
| 63 | 36227←36724 | 165 | Lysin | lysin (165 aa, Phage cdtI) | YP_001272571 | 4e-105, (97/165) |
|  |  |  |  | Endolysin (167 aa, phage SE1) | AAY46497 | 2e-107, (90/165) |
| 64 | 36721←37164 | 147 | Lysis | Bacteriophage lysis protein (147 aa, *E.coli* F11) | ZP_03034679 | 2e-97, (94/147) |
|  |  |  |  | Gene 63 protein (145 aa, phage Sf6) | NP_958237 | 9e-73, (77/145) |
|  |  |  |  | Rz lysis protein (145 aa, phage epsilon34) | YP_002533526 | 7e-72, (77/145） |
| 65 | 37203←37577 | 124 | Unknown | Hypothetical protein HMPREF9553_04187 (124 aa,  *E. coli* MS 200-1) | ZP_07180031 | 7e-75, (88/124) |
| 66 | 37964←38314 | 116 | Restriction endonuclease | HNH endonuclease (116 aa, *E. coli* B088) | ZP_06661876 | 1e-81, (100/116) |
|  |  |  |  | Hypothetical protein SfVp53 (116 aa, phage SfV) | NP_599085 | 5e-78, (95/116) |
