# Supplementary figures and images for: Isolation and genomic characterization of SfI, a serotype-converting bacteriophage of Shigella flexneri
Source: BMC Microbiol. 2013 Feb 17;13:39. doi: 10.1186/1471-2180-13-39 (PMC3636060; doi:10.1186/1471-2180-13-39)

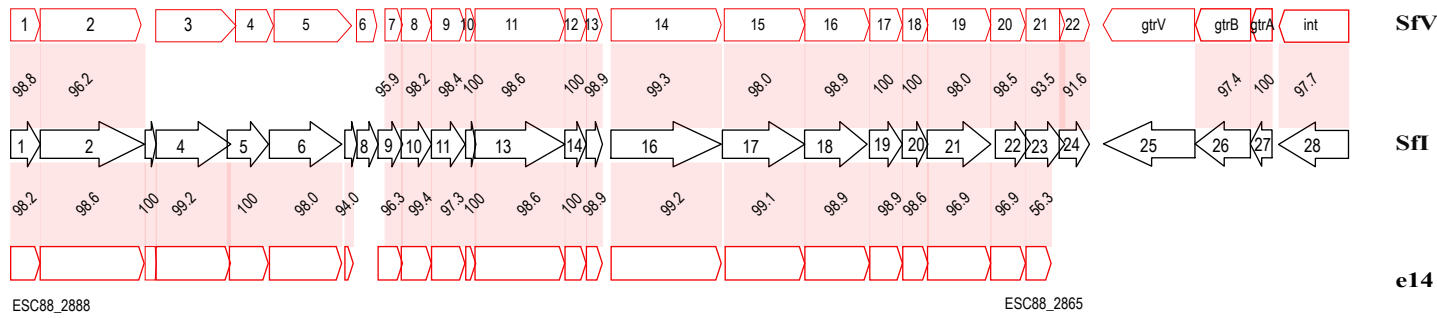

Supplement: Additional file 2: Figure S1 — Gene by gene comparison of homologous regions of SfI with S. flexneri phage SfV and E. coli prophage e14. The arrows indicate the predicted proteins and orientation of the ORFs. The regions marked with a lightly red rectangle represent >50% sequence identity at amino acid level. [file 1471-2180-13-39-S2.pdf]
